# Visualization of Spatiotemporal Energy Dynamics of Hippocampal Neurons by Mass Spectrometry during a Kainate-Induced Seizure

**DOI:** 10.1371/journal.pone.0017952

**Published:** 2011-03-22

**Authors:** Yuki Sugiura, Ryo Taguchi, Mitsutoshi Setou

**Affiliations:** 1 Department of Molecular Anatomy, Hamamatsu Medical School, Higashi-ku, Hamamatsu, Shizuoka, Japan; 2 Department of Metabolome, Graduate School of Medicine, The University of Tokyo, Bunkyo-ku, Tokyo, Japan; Universität Heidelberg, Germany

## Abstract

We report the use of matrix-assisted laser desorption/ionization (MALDI) imaging mass spectrometry combined with capillary electrophoresis (CE) mass spectrometry to visualize energy metabolism in the mouse hippocampus by imaging energy-related metabolites. We show the distribution patterns of ATP, ADP, and AMP in the hippocampus as well as changes in their amounts and distribution patterns in a murine model of limbic, kainate-induced seizure. As an acute response to kainate administration, we found massive and moderate reductions in ATP and ADP levels, respectively, but no significant changes in AMP levels—especially in cells of the CA3 layer. The results suggest the existence of CA3 neuron-selective energy metabolism at the anhydride bonds of ATP and ADP in the hippocampal neurons during seizure. In addition, metabolome analysis of energy synthesis pathways indicates accelerated glycolysis and possibly TCA cycle activity during seizure, presumably due to the depletion of ATP. Consistent with this result, the observed energy depletion significantly recovered up to 180 min after kainate administration. However, the recovery rate was remarkably low in part of the data-pixel population in the CA3 cell layer region, which likely reflects acute and CA3-selective neural death. Taken together, the present approach successfully revealed the spatiotemporal energy metabolism of the mouse hippocampus at a cellular resolution—both quantitatively and qualitatively. We aim to further elucidate various metabolic processes in the neural system.

## Introduction

In the life sciences, transcripts are localized using *in situ* hybridization with oligonucleotide probes, and proteins are localized using immunohistochemical analysis with antibodies. On the basis of microprobe mass analysis which has been used for the localization of atoms [1] and small molecules [2], recently, imaging mass spectrometry (IMS) of biological organic metabolites has been practically utilized [3], [4], [5]. Metabolites, which include metabolic intermediates such as lipids, amino acids, organic acids, and small signaling molecules, are the end products of cellular regulatory processes. Their concentrations can be altered by changes in physiological or pathological conditions; they can regulate various biological phenomena depending on their concentrations. Therefore, metabolites can act as functional entities within cells and tissues. Recent progress in IMS has established a method that enables the simultaneous visualization of a wide range of metabolites with high sensitivity and spatial resolution [6].

At present, 2 ionization methods—secondary ion mass spectrometry (SIMS) and matrix-assisted laser desorption ionization (MALDI)—are widely used for performing IMS for biological [6] and medical samples [5], [7]. An advantage of the SIMS-based IMS is that it offers high spatial resolution (a few hundred nanometers) because of the highly focused primary ion beam for molecular ionization. Another advantage is that interference of matrix cluster ions can be eliminated, because SIMS enables matrix-free ionization, which is particularly useful in the analysis of small molecules. At present, SIMS-based IMS is applied to surface imaging of small molecules in small cells [8], [9]. However, SIMS is a relatively “hard ionization” technique compared to MALDI, and this property limits the analyzable molecules to only a part of ions over a narrow *m*/*z* range (*m*/*z*<1,000).

On the other hand, MALDI-IMS can measure ions over a wide mass range (from *m/z* of several hundreds to several ten thousands). Owing to the its “soft ionization” property, a variety of studies thus far have applied MALDI-IMS for visualization of molecules, from small metabolites [10], [11], [12] to much larger proteins [13], [14], in the biological/medical samples. In addition, MALDI-IMS can be applied for molecular identification via detailed structural analysis by tandem mass spectrometry (MS*^n^*) [15]. At present, MALDI-IMS involves several types of MALDI-ion sources coupled with advanced mass analyzers and enables complete analysis of metabolites in tissue sections [16], [17]. However, at present, a limitation is that the spatial resolution of MALDI instruments, which is determined by the spot diameter of the MALDI laser (10–100 µm), is much lesser than that of SIMS.

In the present study, we report the results of using this MALDI-based imaging technique to visualize energy metabolism in the mouse hippocampus via the imaging of energy-related metabolites. Cellular metabolic processes utilize ATP as an energy source, thereby converting it to ADP or AMP. Therefore, techniques for the simultaneous visualization of these adenosine nucleotides provide valuable information regarding the energy production and consumption in the tissues. We studied the changes in the distribution and amounts of such energy-related metabolites under neural stimulation within a short time scale *in vivo*. In order to do so, we used a murine model of kainate-induced limbic seizure. Kainate is an analogue of glutamate, and its administration causes severe seizures primarily observed at 30 minutes after administration [18]; it also causes some rather selective neuronal effects, including cell losses, gliosis [19], and mossy fiber sprouting [20], particularly in CA3 regions in the hippocampus. In this study, we visualized CA3–cells selective energy consumption during seizures as well as the accelerated production of energy to compensate for the depletion. Furthermore, high-resolution imaging analysis revealed that partial populations of the CA3 cells failed to recover after the energy depletion, presumably reflecting acute neuronal degradation. Since we were able to visualize heterogeneous responses of the cells in the microtissue region to kainate with respect to energy consumption and recovery at cellular resolution *in vivo*, the presented approach is a powerful tool for studying the mechanisms behind selective kainate toxicity in CA3 neurons. In the future, we aim to further elucidate various metabolic processes in the nervous system.

## Results and Discussion

### Massive consumption of adenosine nucleotides in CA3 cell layer during kainate-induced seizure

**Figure 1:**
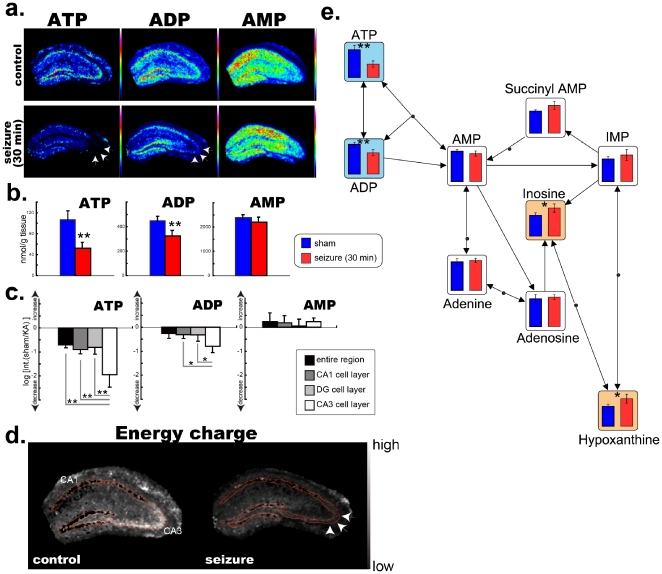
CA3 cell-selective consumption of ATP and ADP during a kainate-induced seizure. **a**. MALDI imaging of adenosine nucleotides in a mouse hippocampus. **b**. Absolute quantification of ATP, ADP, and AMP in a mouse cerebrum using CE-MS. Massive reductions in the levels of ATP and ADP, but not AMP were observed during kainate-induced seizures. (n = 4 mice for each group; *p<0.05, **p<0.01; Welch's *t*-test). **c**. Results of the relative quantification of ion intensity for ATP, ADP, and AMP calculated from the averaged mass spectra of each hippocampal sub-region obtained using MALDI imaging. The values shown are logarithmic ratios of ion intensities between sham-operated (sham) and kainate-treated mice (KA). (n = 4 mice for each group; *p<0.05, **p<0.01; Welch's *t*-test). **d**. Mapping of energy-charge index values on tissue sections. The region-specific reduction of these values in the CA3 region (arrows) suggests massive energy metabolism in CA3 neurons. **e**. Relative quantitative comparison of adenosine nucleotides and related metabolites using CE-MS. Each result is mapped on the metabolic pathway and clearly shows the depletion of ATP and ADP due their conversion into downstream metabolites. Colored graphs indicate significant increases (orange) and decreases (blue). (n = 4, *p<0.01, **p<0.05; Welch's *t*-test).

First, we determined the distribution patterns of ATP, ADP, and AMP in the basal state of the mouse hippocampus by performing MALDI imaging with 9-aminoacridine as the matrix [21] (Fig. 1A, upper panels). It is interesting to note that ATP and AMP were localized in the cell-layer regions of the hippocampus, whereas AMP was widely distributed among dendritic regions of the hippocampus. Next, we proceeded to study the changes in the distribution and amounts of these energy-related metabolites under neural stimulation in a short time scale (control vs. 30 min after stimulation). To do so, we used a murine model of kainate-induced limbic seizure. The administration of kainate causes severe seizures that are primarily observed at 30 minutes after administration [18]. MALDI imaging during such acute seizures (at 30 minutes) showed massive and moderate reductions in ATP and ADP levels respectively (*p*<0.01 and *p*<0.05, n = 4 for each group); there were no significant changes in AMP levels, suggesting enhanced energy consumption at the anhydride bonds of ATP and ADP during the seizure (Fig. 1a, lower panels). In particular, cells in the CA3 layer, which are particularly rich in neurons that express kainate receptors [22], [23], exhibited massive reductions in ATP and ADP levels (arrows); ATP was reduced by approximately 75% within 30 minutes after administration. In addition, we validated the quantitative changes obtained by MALDI imaging using CE-MS-based metabolomics [24]. Due to its high quantitative ability and wide molecular coverage, the CE-MS technique is helpful for quantitative validation [25]. Absolute quantifications of ATP, ADP, and AMP in the mouse cerebrum were performed by CE-MS and revealed massive reductions in the levels of ATP and ADP (both *p*<0.005), but not AMP during the seizure (Fig. 1b and Table 1).

**Table 1:** Results of quantitative CE-MS metabolomics comparing 27 metabolites which showed significant change between control and kainate-treated mouse brain extracts.

	Concentration (nmol/g)^†^, n = 4						
	Control group		Kainate group				
metabolite	Mean	S.D.	Mean	S.D.	ratio	t-test	
3-Phosphoglyceric acid	80.8	13.4	33.9	6.2	**0.42**	0.001	**
Fructose 6-phosphate	7.0	0.7	3.0	0.4	**0.44**	0.0001	***
ATP	106	17	52	12	**0.49**	0.002	**
Fructose 1,6-diphosphate	115	15	65	13	**0.56**	0.003	**
Glucose 6-phosphate	36.5	5.7	22.7	1.8	**0.62**	0.004	**
Ornithine	4.6	0.9	3.0	0.2	**0.65**	0.011	*
Dihydroxyacetone phosphate	13.2	2.1	8.8	2.0	**0.67**	0.021	*
UDP	9.1	1.8	6.2	0.7	**0.68**	0.024	*
CDP	3.6	0.6	2.5	0.5	**0.69**	0.031	*
ADP	448	36	323	46	**0.72**	0.005	**
3-Hydroxybutyric acid	42.2	6.8	33.4	1.3	**0.79**	0.044	*
GTP	48.3	5.9	38.4	4.6	**0.79**	0.037	*
*S*-Adenosylmethionine	17.1	1.0	13.8	0.6	**0.81**	0.001	**
Asn	92.4	3.4	80.4	0.8	**0.87**	0.0004	***
Leu	65.0	1.3	59.3	2.9	**0.91**	0.011	*
Gln	3882	26	4012	76	**1.03**	0.018	**
Thr	221	7	251	5	**1.14**	0.0004	***
*cis*-Aconitic acid	6.2	0.2	7.6	1.0	**1.22**	0.032	*
Choline	139	14	176	13	**1.27**	0.008	**
Ala	582	6	778	120	**1.34**	0.017	*
Inosine	142	19	193	28	**1.36**	0.024	*
Hypoxanthine	83.5	9.6	116.4	18.9	**1.39**	0.021	*
Betaine aldehyde	0.30	0.04	0.44	0.06	**1.46**	0.010	*
Acetyl CoA	1.4	0.2	2.1	0.2	**1.48**	0.003	**
Trp	7.8	0.9	12.2	1.0	**1.56**	0.001	**
2-Hydroxybutyric acid	4.4	0.7	7.7	1.0	**1.74**	0.002	**
Cys	2.0	0.3	4.1	1.1	**2.06**	0.008	**

The full name of each abbreviated metabolite name is shown in Table S1.

Next, to visualize the two-dimensional information regarding energy metabolism, we calculated the energy-charge index for each pixel (i.e., data point); this index represents the amount of stored cellular energy and was calculated as follows: 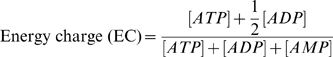 [26]

According to this equation, a high index value indicates a high proportion of ATP and ADP, and a low proportion of AMP among the adenosine nucleotides. Furthermore, we constructed pseudocolor maps of this index that clearly show enhanced metabolic turnover in the CA3 subfield (Fig. 1d, arrows). The characteristic high energy-charge index in the CA3 cell layer observed in the hippocampus of the control mice almost completely disappeared after the administration of kainate; this suggests the presence of distinct energy metabolism at the anhydride bonds of ATP and ADP in the hippocampal neurons during the seizure. MALDI imaging and CE-MS were also performed for other nucleotides—namely guanosine and uridine nucleotides—and revealed that among all 3 nucleotides, adenosine nucleotide exhibits the most characteristic and prominent cellular dynamics (Fig. S1). Furthermore, consumed ATP and ATP appeared to be metabolized not only to AMP, but also to further downstream nucleotides—namely IMP, inosine, and hypoxantine—reflecting rather severe degradation of energy-storing metabolites (Fig. 1d).

Kainate stimulation rapidly activates the various ATP-consuming pathways in neurons [27], and the observed CA3-selective energy depletion can be accounted for by the strong affinity of kainate in the CA3 region [23]. The rich kainate receptors there [22], [23] as well as trans membrane AMPA associated protein (TARP) associated AMPA receptors, which also excite hippocampal neurons efficiently and have critical role in kainate-induced neuronal toxicity, could be stimulated under presence of kainate [28], and for both, CA3 specific neural circuit structure *in vivo*, i.e., recurrent excitation could magnify the stimulation in region specific manner [29]. Under such stimulation, acute ATP depletion would primarily result in the inactivation of Na-K^+^ pumps, because such Na^+^/K^+^-ATPases are the most energy-consuming pathway in neurons [30]; this in turn causes the disruption of intracellular ion homeostasis. Repeated or prolonged depolarization as a result of such disrupted ion homeostasis can eventually lead to excitotoxicity; in this case, excitotoxicity is likely to occur in the CA3 hippocampal region, resulting in a massive intracellular calcium influx and leading to the activation of Ca^2+^ dependent phospholipases, the irreversible disruption of the membrane, and cellular lysis [22]. The observed massive reductions in ATP level (Fig. 1a) as well as the EC index (Fig. 1d) were remarkably greater than in the other hippocampal regions. For example, the reduction in ATP levels was 2.5 and 1.4 times greater than in the whole hippocampus and CA1 cell-layer region respectively. Therefore, this could be one of the primary reasons for the particular vulnerability of CA3 to enhanced neuronal discharge produced by kainate—particularly for the nuronal degradation observed during the early phase of kainate-induced seizures [31], [32].

### Accelerated energy production pathways compensate for ATP depletion during seizure

**Figure 2:**
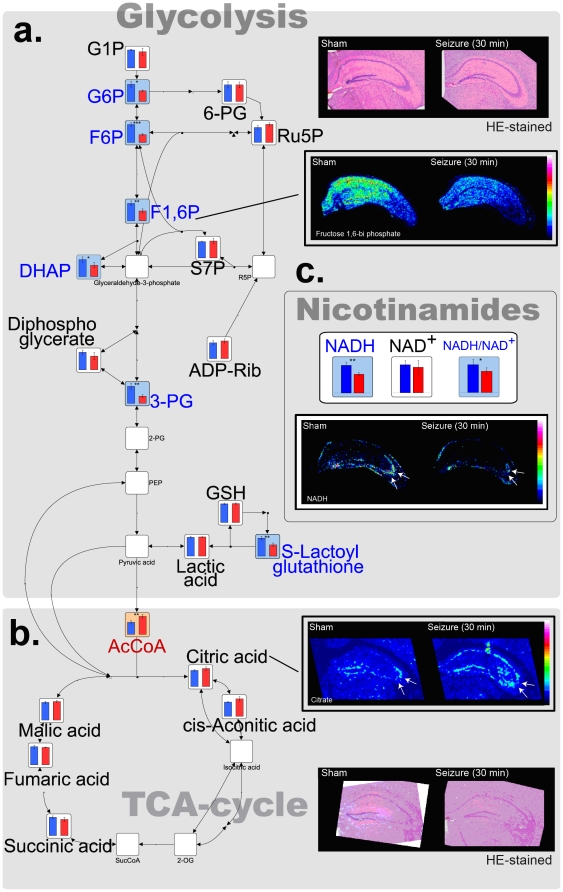
Enhanced energy production pathways compensate for ATP consumption. Relative quantitative comparisons of glycolysis and TCA cycle-related metabolites using CE-MS. In addition, IMS results of key metabolites for each pathway, including fructose 1,6-bi-phosphates in glycolysis and citrate in the TCA cycle, are also presented. Each result is mapped on the metabolic pathway (n = 4; *p<0.05, **p<0.01; Welch's *t*-test). Colored graphs indicate significant increases (orange) and decreases (blue). Open graphs indicate “not detected.” The full name of each abbreviated metabolite is shown in Table S1.

Analyses of important energy ‘production’ pathways, including the glycolysis pathway and tri-carboxylic acid (TCA) cycle, revealed their acceleration, presumably in response to ATP depletion. Fig. 2A shows the metabolic snapshots of the glycolysis pathway indicating enhanced glycolytic activity as an initial response to kainite treatment; most of the glycolytic intermediates were significantly decreased 30 min after kainate administration (blue-colored graphs), suggesting the occurrence of acute glycolysis. While considering previous studies reporting enhanced glucose uptake in the hippocampus—especially the CA3 region in response to kainate-injection [18], [33]—our data indicate an accelerated molecular flux of the glycolytic pathway to downstream pathways, which in turn enhances the production of ATP and NADH, presumably to compensate for the aforementioned ATP depletion.

Another important finding of the present study is that lactate did not exhibit any significant changes, therefore indicating that anaerobic respiration did not accelerate at this time point. It is well known that when anaerobic respiration is dominant, (e.g., under ischemic conditions) lactate is increasingly produced from pyruvate and subsequently accumulates [25]. Therefore, in the presented early time points of kainate-induced seizures, it is reasonable to suggest that the increased molecular flux of the glycolytic pathway was then transferred into the TCA cycle for further aerobic respiration via acetyl-CoA in the mitochondrial organization. Actually, a significant increase in acetyl-CoA (*p*<0.01, n = 4 for each group; 1.5-fold increase; orange-colored graph), and increases in citrate and *cis*-aconitic acid (both show 1.2-fold increases; Table 1) were observed (Fig. 2b); this appears to imply that increased influx into the TCA cycle. Furthermore, the results of MS/MS imaging confirmed that the increase in citrate primarily occurred in the CA3 region (Fig. 2b, arrows).

To further determine the presence of an active TCA cycle, we also studied changes in NADH and NAD^+^; the ratio between these dominantly affects mitochondrial TCA cycle activity [34], [35]. NADH and the NADH/NAD^+^ ratio were significantly reduced by 32% and 24% respectively (*p*<0.01 and *p*<0.05, n = 4 for each group) (Fig. 2c); imaging revealed that these decreases occurred especially in cells of the CA3 layer, (Fig. 2c, arrows), suggesting CA3 neuron selective reduction. Since both measures are known to increase when the TCA cycle is blocked [25], the observed reductions in NADH and NADH/NAD^+^ ratio suggest active oxidative phosphorylation via the TCA cycle at this time point. Our assumption is consistent with previously reported increases in hippocampal blood flow and CO_2_ pressure and a reduction of oxygen pressure observed 30 min after kainate injection, which indicate accelerated aerobic respiration as an initial response to kainate [32]. Together with the data shown in Fig. 2, it is reasonable to suggest that the occurrence of active or even accelerated energy production through glycolysis fueled by the consumption of glycolytic intermediates as well as oxidative phosphorylation via the TCA cycle by the consumption of NADH, compensates for the acute and large degree of ATP consumption as an acute response to kainate—especially in CA3 neurons.

### Recovery of the depleted-energy state in cells in CA1 and CA3 regions within 3 hours

**Figure 3:**
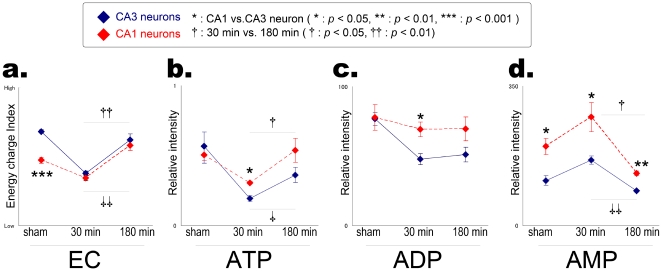
Recovery of energy consumption in CA1 and CA3 cells. The MALDI-imaging results of mouse hippocampus of control and kainate-administered mice at 30 and 180 minutes after administration. The signals of adenosine nucleotides as well as the calculated EC-index values on each pixel, were collected from CA1 and CA3 cell-layer regions and averaged. Values are presented as means (S.E.M.) Control (sham-operated, n = 3), 30 min (n = 4), and 180 min (n = 4).

Next, we determined whether the observed energy consumption was recoverable—particularly in cells in the CA1 and CA3 layers. This was done because knowing this would increase the understanding of whether the presented energy depletion is correlated with the observed CA3-selective neuronal death by kainate *in vivo*. In order to do so, we performed time-course MALDI imaging of mouse hippocampus on both control and kainate-administered mice at 30 and 180 minutes after administration. The signals of adenosine nucleotides as well as the calculated EC-index values on the each pixel were collected from the CA1 and CA3 cell-layer regions and averaged (Fig. 3). The results revealed that the acute energy depletion that was mainly observed in CA3 rather than in CA1 cells and was a reversible phenomenon in the both cell layers.

At their basal state, cells in CA3 exhibited significantly greater EC values than those of CA1 (*p*<0.001, n = 4 for each group, Fig. 3a) mainly due to lower AMP levels (Fig. 3d). As acute responses to kainate administration, both the decreased rates of ATP and ADP, and the increased rate of AMP after 30 min were significantly higher in CA3 than in CA1 cells. Interestingly, at 180 min after administration, both CA1 and CA3 cells exhibited a significant recovery in energy consumption—namely, increases in ATP and decreases in AMP. In particular, CA1 cells demonstrated higher recovery rates of adenosine nucleotides than CA3 cells, resulting in a clear recovery in the EC index; this value was even higher than those of the control mice (Fig. 3a).

The observed recoveries imply that accelerated energy production compensates for the energy depletion caused by kainate stimulation, thus supporting our assumption presented in Fig. 2 discussion. However, it is worth noting that CA3 cells did not recover to the same level as those in the control mice. In particular, ADP levels remained at only 66% of the intensity of the control mice (*p*<0.01, n = 4 for each group). On the other hand, CA1 cells recovered to almost the same energy conditions of the control mice.

### Heterogeneous energy recovery capacity within cells in the CA3 cell layer

**Figure 4:**
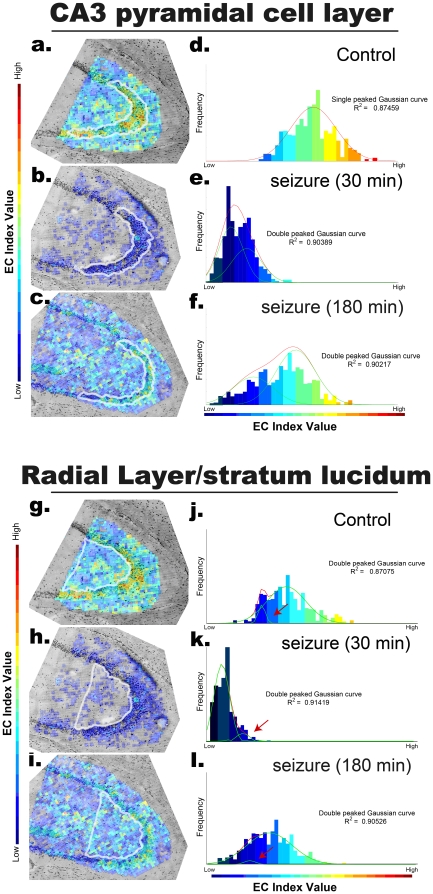
Heterogeneous energy recovery capacity within CA3 cell populations. Two-dimensional EC-value maps were reconstructed from MALDI-imaging data (at a spatial resolution of 22 µm) by calculating EC values at each pixel using adenosine nucleotide signals; these are shown in optical images of HE-stained tissue sections (a–c). The pixel appearance frequencies of each EC-index value range are displayed as histograms (d–f). The histograms were fitted into single- and double-peaked Gaussian distribution curves; for the 3 presented conditions, the curve with greatest correlation factor (R2) was adopted and shown.

The detailed analyses of energy-state changes of CA3 cells revealed a heterogeneous energy recovery capacity within CA3 cells populations (Fig. 4). Here, we show two-dimensional EC-value maps on optical images of HE-stained tissue sections (Fig. 4a–c, g–i); changes in the appearance frequency of pixels in each EC-index value are displayed as histograms calculated from the time-course MALDI imaging (Fig. 4c–f, j–l). Since the scan pitch was set at 22 µm (similar in size to a few CA3 neurons), the pixel appearance frequency (Y-axis) can be interpreted as CA3 neuron appearance frequency in each EC-index value range. As seen at the basal state, the CA3 cell layer shows remarkably high EC values (Fig. 4a), and the histogram of EC-index values exhibits a Gaussian distribution (fitted to a single-peaked curve, R2 = 0.87) in the relatively higher EC-value range (Fig. 4d). At 30 min after kainate administration, pixels with higher EC values completely disappeared from the CA3 region (Fig. 4b). Reflecting this reduction, the entire part of the histogram shifted towards lower EC-values where it could be better fitted into a double-peaked Gaussian curve than a single-peaked one. This means that there were 2 populations of data pixels in the CA3 cell layer that have different sensitivities to kainate (Fig. 4e). At 180 min after kainate administration, most of the pixels returned to having mid-ranged EC values (Fig. 4c). However, partial populations of CA3 pixels were still observed at low EC values (Fig. 4f). In fact, the histogram was also better fitted to a double-peaked Gaussian curve by subdividing the CA3 region-derived pixels into a dominant population that shows a large recovery and a sub-population with a lower recovery capacity. Such heterogeneity caused the histogram to have a planular shape. In contrast, CA3 regions of outside of the cell layer containing radial layer and stratum lucidum (Fig. 4g–i) showed severe EC-index reduction (Fig. 4k), but almost complete recovery at 180 min after dose (Fig. 4l). We additionally note that a small population which seemed to be insensitive to kainate could be observed in this region (red arrows).

In the present study, we suggest the possibility that CA3-selective and massive ATP depletion within 30 min after dose contributes to acute necrotic degradation—especially for partial CA3 cell populations that exhibit severe energy depletion (Fig. 4f). Previous studies report that the kainate-induced preferential degradation of CA 3 neurons at various time points—generally within several hours [33], [36], tens of hours, and days after dose [31], [32], [36]—and the underlying toxic mechanism are explainable by both apoptotic pathways [31] as well as the necrotic one [36]. However, since it takes several hours to go through the neural apoptotic pathway [37], [38], [39], the toxic mechanism behind acute and late-onset neural degradation might be different, and the pathological features at different phases might be described distinctly. In the present study, with respect to the energy state, we distinguished the acute phase, in which early energy depletion (Fig. 1) and the acceleration of energy production (Fig. 2) were observed, from the latter phase (3 hours after kainate administration), in which the cellular energy state showed significant recovery (Fig. 3). In addition to such time-dependent changes in energy state, while taking advantage of high-resolution imaging analyses, we found different kainate responses between CA1 and CA3 cells, and among CA3 cell populations (i.e., spatially dependent energy changes) (Fig. 4). Although cllasical studies demonstrate kainate-induced ATP degradation using cultured neurons [40], such *in vitro* observations cannot distinguish hippocampal cell types or sub-populations of the same cell type. In this study, both the time-course and spatially resolved analyses *in vivo* indicate that the energy depletion is limited to some CA3 neurons at the acute phase; therefore, the observed effect of the energy degradation might be larger than when studying with cultured neurons. Due to these reasons, it is not unreasonable to suggest that acute CA3 neuron-selective death may be attributed to the necrotic pathway primarily due to the observed energy depletion.

From the methodological aspect, IMS has several important advantages for the assessment of dynamic changes in the amount of metabolites in small tissue regions. Researchers could employ physical separation procedures to dissect small tissue regions, such as laser capture microdissection technology, however, IMS has several important advantages over such physical separation procedures, which we consider to be important, especially for metabolites with rapid turnover rates. These advantages are as follows: (i) Energy metabolites with rapid metabolic turnover rates, such as ATP and ADP, would be degraded during the physical separation process that lasts several minutes at room temperature. Unthawed tissue sections should not be left at room temperature in order to prevent them from undergoing postmortem degradation. In this regard, IMS could minimize such degradation during sample preparation, because matrix containing an organic solution was immediately sprayed on unthawed tissue sections. The matrix solvent applied to the tissues could suppress most of the postmortem enzymatic activities by denaturing the enzymes; furthermore, the metabolites were extracted from tissues and co-crystallized with the matrix on the sample surface. Therefore, owing to the simple and fast sample preparation procedures, MALDI-IMS has an important advantage because it minimizes postmortem metabolite degradation, compared to the physical separation procedures coupled with biochemical analyses. (ii) Another advantage of IMS is that because it is a type of a molecular imaging technique, it can yield unexpected results that otherwise cannot be obtained by analysis of dissected tissues only from predefined regions. As shown in Fig. 4, we found that the data pixels in CA3 cell layer region consisted of 2 different clusters, depending on changes in energy metabolism in response to the kainate administration. This result could be obtained only by the imaging experiment.

In conclusion, we obtained images reflecting the metabolic status of mouse hippocampal cells. In the future, we aim to further uncover various spatiotemporal metabolic processes in the nervous system by using IMS coupled with CE-MS technology.

## Materials and Methods

### Kainate administration

All experiments on mice were conducted according to protocols approved by the Animal Care and Use Committee of the Hamamatsu School of Medicine. Eight-week-old male C57BL/6J mice were intraperitoneally injected with kainate (25 mg/kg body weight, in saline) and then sacrificed at the indicated time points (n = 4 for each group). The treated animals were rated for seizure severity on the basis of a previously defined rating scale [41]; those with scores less than 5 were used for analysis.

### Sacrifice of animals and tissue extraction

Brain is susceptible to postmortem changes in contents of cerebral metabolites, thus care should be paid to minimize autolytic changes of metabolites. To achieve this, traditionally, *in-situ* freezing method [42], [43], which enables to lower the tissue temperature while maintain blood flow during the freezing process under anesthesia was usually employed [25]. However, in present study, since kainate-treated mice had severe and conclusive seizure, therefore we could not fix mice for anesthesia. Because of the reason, alternatively, we here strictly controlled time for brain extraction; mouse brains were extracted within 1 minute (typically 40 seconds) after sacrifice. The trimmed tissue blocks were immediately frozen in powdered dry ice (allowing tissues to be frozen without cracking) and stored at −80°C until use. Due to the large metabolic changes between control and kainate-treated mice as shown, here we obtained reproducible and significant results, however, we note that despite of the short handling time, the detected brain adenylate levels were seemed to be degraded when compared *in-situ* freezing method [25].

### Tissue section preparation

Tissues blocks were sectioned at −16°C using a cryostat (CM 3050; Leica, Germany) to a thickness of 5 µm, as described in previous reports [44]. Although brain blocks were held by an optimum cutting temperature (OCT) polymer, they were not embedded into it, because it was thought that any residual polymer on the tissue slices might degrade the mass spectra [45]. The frozen sections were thaw-mounted on indium-tin-oxide (ITO)-coated glass slides (Bruker Daltonics).

### Sample preparation and MALDI imaging

Tissue preparation and MALDI-IMS were performed using 9-aminoacridine as the matrix (10 mg/mL, dissolved in 70% methanol). Matrices were simultaneously applied to the tissue sections in order to maintain consistent analyte extraction and co-crystallization conditions. MALDI imaging was performed using an Ultra Flex 2 MALDI-time-of-flight (TOF) mass spectrometer (Bruker Daltonics, Leipzig, Germany) equipped with a Nd:YAG laser. Data were acquired in the negative reflectron mode. Each spectrum was the result of 100 laser shots at each data point. In this analysis, signals between *m/z* 100 and 1000 were collected. The interval between data points was 20 µm; in total, we obtained approximately 6000 data points for each hippocampal region. Image reconstruction was performed using FlexImaging 2.0 (Bruker Daltonics).

### MS/MS and MS/MS imaging

Molecular identification was performed with LTQ-XL (Thermo Fisher Scientific) equipped with an intermediate-pressure MALDI ion source. All MS/MS spectra were shown in Fig. S2 in the supporting information. Since the ions at *m/z* 191 corresponding to citrate contain multiple ions within the same nominal mass, we performed MS/MS imaging with LTQ-XL at a raster scan pitch of 40 µm. Image reconstruction was performed using Image Quest (Thremo Fisher Scientific).

### Data analysis

Energy Charge calculation was performed by in-house software constructed by MATLAB™ software (Mathworks, Inc., Sherborn, MA, USA). Creation of histograms presented Fig. 4 and their fitting to Gaussian profile were performed by Origin 8.0 software (Microcal Software Inc., Northampton, Massachusetts, USA).

### CE-MS-based metabolomics

Frozen mouse brains were homogenized in methanol (500 µL/100 mg tissue) using a beads homogenizer (Micro Smash MS-100R; Tomy, Tokyo, Japan), followed by the addition of an equal volume of chloroform and 0.4 times the volume of Milli-Q water. After centrifugation (3 cycles at 4,000 rpm for 60 s), the aqueous phases were ultrafiltered using an ultrafiltration tube (Ultrafree-MC, UFC3 LCC; Millipore, USA) and the filtrates were dried. The dried residues were redissolved in 50 µL Milli-Q water and were used for CE-MS.

CE-MS experiments were performed using Agilent CE systems equipped with a time-of-flight mass spectrometer (TOF-MS) and a built-in diode-array detector (Agilent Technologies). Cationic metabolites were analyzed with a fused-silica capillary (50 µm i.d. ×80 cm total length) with cation buffer solution (Human Metabolome Technologies) as the electrolyte. The samples were injected at a pressure of 5.0 kPa for 10 s (approximately 10 nL). The applied voltage was set at 30 kV. Electrospray ionization-mass spectrometry (ESI-MS) was conducted in the positive ion mode, and the capillary voltage was set at 4,000 V. The spectrometer was scanned from m/z 50 to 1,000. Other conditions were the same as in the cation analysis [46].

Anionic metabolites were analyzed with a fused-silica capillary (50 µm i.d. ×80 cm total length), with anion buffer solution (Human Metabolome Technologies) as the electrolyte.

The samples were injected at a pressure of 5.0 kPa for 25 s (approximately 25 nL). The applied voltage was set at 30 kV. ESI-MS was conducted in the negative ion mode, and the capillary voltage was set at 3,500 V. The spectrometer was scanned from m/z 50 to 1,000. Other conditions were the same as in the anion analysis [47].

Metabolites in the samples were identified by comparing the migration time and m/z ratio with authentic standards, and differences of ±0.5 min and ±10 p.p.m. were permitted respectively and quantified by comparing their peak areas with those of authentic standards using ChemStation software (Agilent Technologies).

## Supporting Information

Figure S1 **Analyses of dynamic changes in guanosine and uridine nucleotides during kainate-induced seizures.** Visualization of the energy-charge index values by MALDI imaging, and absolute quantification by CE-MS for guanosine (A) and uridine (B) nucleotides. Due to their low concentration, cytidine nucleotides could not be detected by either method.
(DOC)

Figure S2 **Results of MS^2^ structural analysis of metabolite ions, corresponding to the ATP, ADP, AMP, fructose bi-phosphate, NADH, and citrate.** Data were obtained with LTQ-XL (Thermo Fisher Scientific) equipped with an intermediate-pressure MALDI ion source.
(DOC)

Table S1 (DOCX)
