# Supplementary material for: Visualization of Spatiotemporal Energy Dynamics of Hippocampal Neurons by Mass Spectrometry during a Kainate-Induced Seizure
Source: PLoS One. 2011 Mar 22;6(3):e17952. doi: 10.1371/journal.pone.0017952 (PMC3062556; doi:10.1371/journal.pone.0017952)
Supplement: Figure S1 — Analyses of dynamic changes in guanosine and uridine nucleotides during kainate-induced seizures. Visualization of the energy-charge index values by MALDI imaging, and absolute quantification by CE-MS for guanosine (A) and uridine (B) nucleotides. Due to their low concentration, cytidine nucleotides could not be detected by either method. (DOC) [file pone.0017952.s001.doc]

　
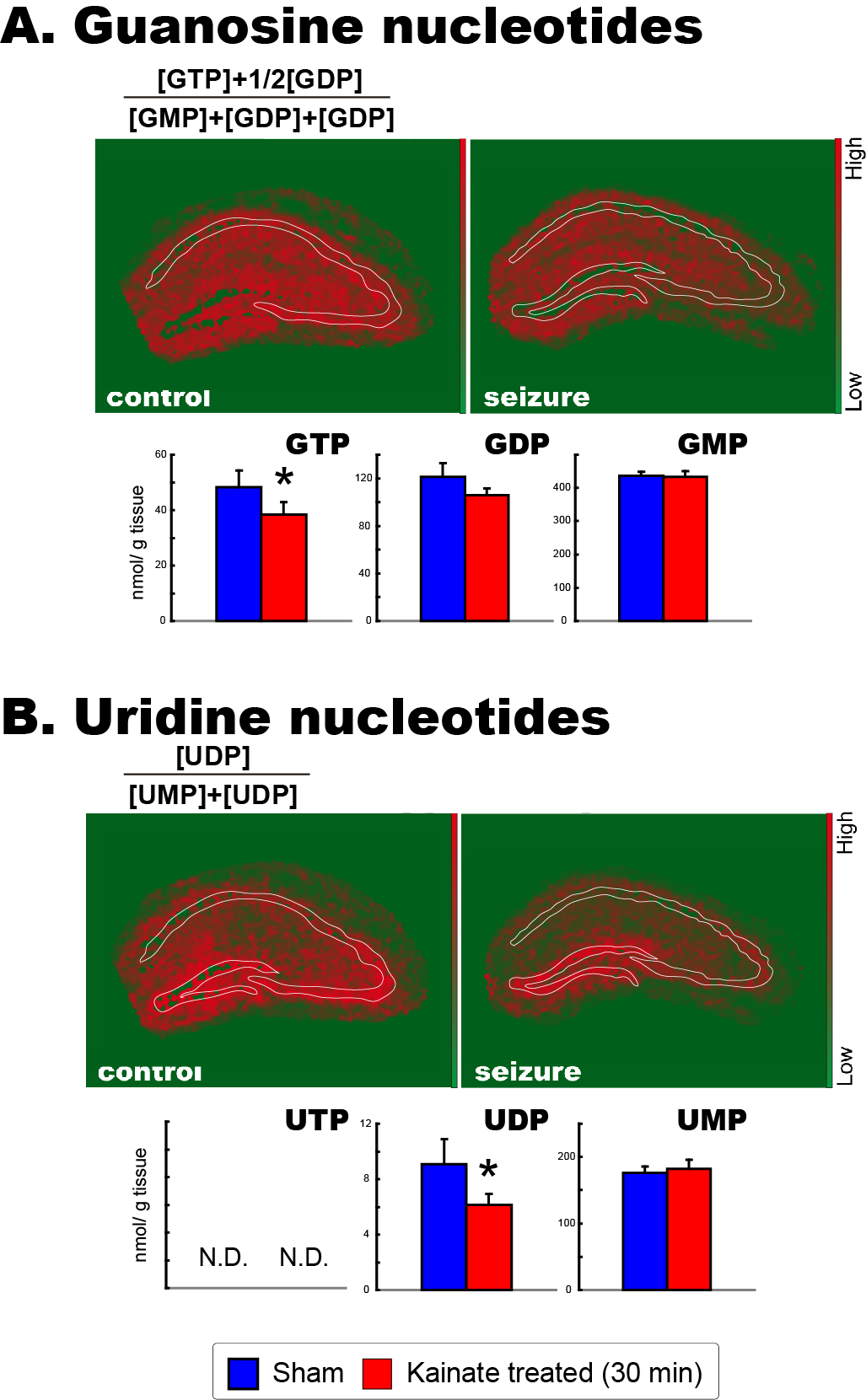


**Fig. S1. Analyses of dynamic changes in guanosine and uridine nucleotides during kainate-induced seizures**

Visualization of the energy-charge index values by MALDI imaging, and absolute quantification by CE-MS for guanosine (A) and uridine (B) nucleotides. Due to their low concentration, cytidine nucleotides could not be detected by either method.
