# Supplementary material for: Visualization of Spatiotemporal Energy Dynamics of Hippocampal Neurons by Mass Spectrometry during a Kainate-Induced Seizure
Source: PLoS One. 2011 Mar 22;6(3):e17952. doi: 10.1371/journal.pone.0017952 (PMC3062556; doi:10.1371/journal.pone.0017952)
Supplement: Figure S2 — Results of MS2 structural analysis of metabolite ions, corresponding to the ATP, ADP, AMP, fructose bi-phosphate, NADH, and citrate. Data were obtained with LTQ-XL (Thermo Fisher Scientific) equipped with an intermediate-pressure MALDI ion source. (DOC) [file pone.0017952.s002.doc]

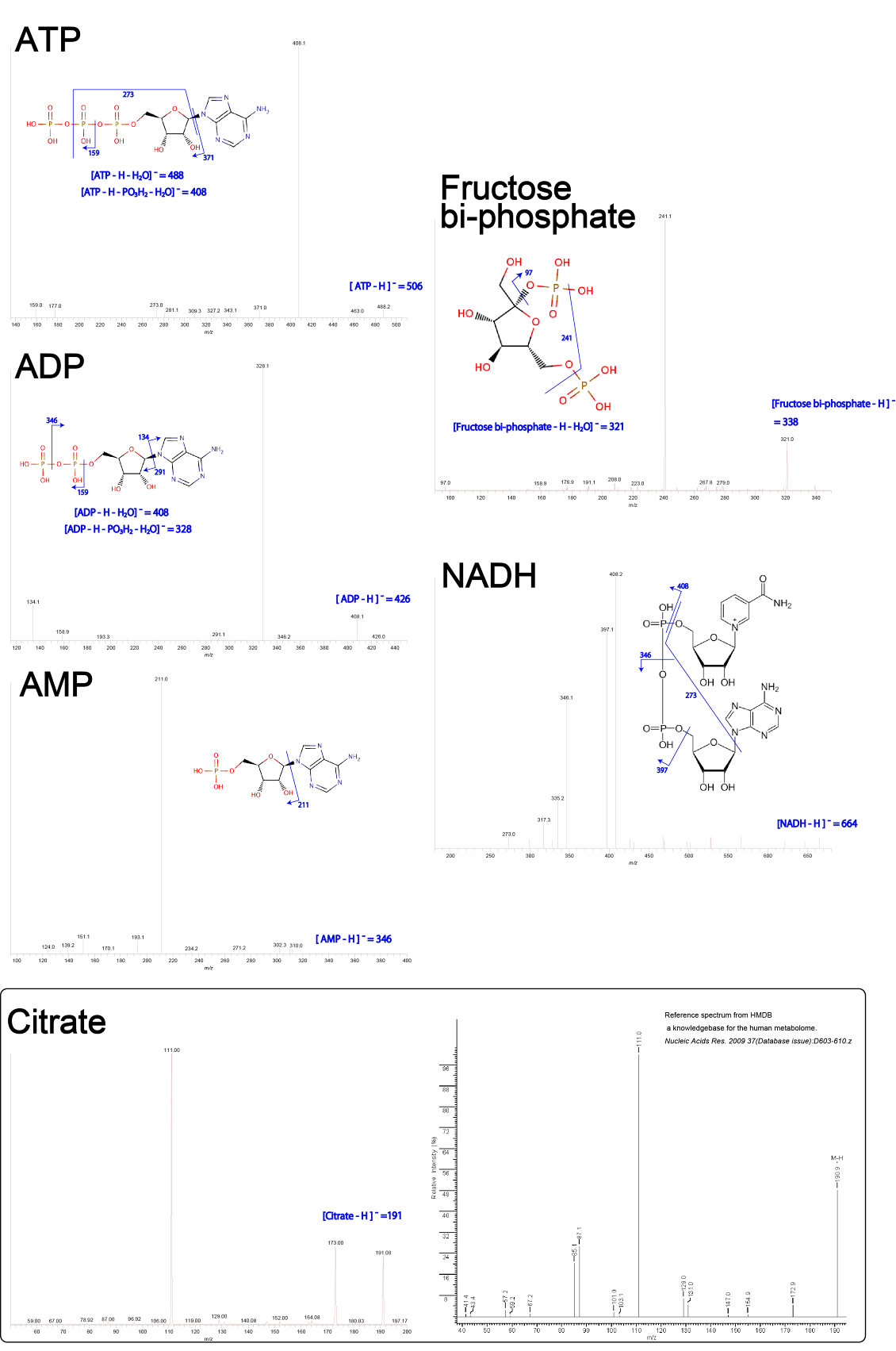


**Fig. S2.** Results of MS^2^ structural analysis of metabolite ions, corresponding to the ATP, ADP, AMP, fructose bi-phosphate, NADH, and citrate. Data were obtained with LTQ-XL (Thermo Fisher Scientific) equipped with an intermediate-pressure MALDI ion source.
