# Supplementary material for: Visualization of Spatiotemporal Energy Dynamics of Hippocampal Neurons by Mass Spectrometry during a Kainate-Induced Seizure
Source: PLoS One. 2011 Mar 22;6(3):e17952. doi: 10.1371/journal.pone.0017952 (PMC3062556; doi:10.1371/journal.pone.0017952)
Supplement: Table S1 — (DOCX) [file pone.0017952.s003.docx]

**Table. S1: Abbreviations of metabolite names**
